# Venous Hemodynamic Dysfunction and Recurrent Miscarriage: Case Series and Literature Review

**DOI:** 10.3390/jcdd12050193

**Published:** 2025-05-18

**Authors:** Elisa Sabattini, Helena Van Kerrebroeck, Wilfried Gyselaers

**Affiliations:** 1 Department Obstetrics and Gynecology, Fondazione IRCCS Ca’Granda—Ospedale Maggiore Policlinico, 20122 Milano, Italy; elisa.sabaz@gmail.com; 2 Department Gynecology and Obstetrics, Ziekenhuis Oost Limburg, 3600 Genk, Belgium; helena.vankerrebroeck@zol.be; 3 Limburg Preeclampsia Clinic & Research Project, Hasselt University, 3500 Hasselt, Belgium; 4 Faculty of Medicine and Life Sciences, Hasselt University, 3500 Hasselt, Belgium

**Keywords:** venous hemodynamics, preconception, recurrent miscarriage, venous Doppler, congestion

## Abstract

(1) Background: Maternal venous hemodynamic dysfunction is an intrinsic part of the pathophysiology of pre-eclampsia and fetal growth restriction. The aim of this study is to evaluate whether venous hemodynamic dysfunction is present in women with a history of (unexplained) recurrent miscarriage, and to link this pilot observation to reported data in the literature. (2) Methods: A retrospective search of hospital records was conducted to find data on recurrent miscarriage and hemodynamics assessment prior to conception. We also performed a scoping search of the literature regarding the association between recurrent miscarriage and maternal hemodynamics, reproductive outcomes, maternal complications, neonatal complications, and long-term cardiovascular function in women and their offspring. (3) Results: Six out of nine women with a history of recurrent miscarriage had preconception venous hemodynamic dysfunction. This observation is in line with the reported data on reduced venous reserves in association with low plasma volume in women with recurrent miscarriage, and adds to the reported link between recurrent miscarriage, poor reproductive outcomes, and chronic cardiovascular disease. (4) Discussion: This retrospective observational cohort supports an association between venous hemodynamic dysfunction and recurrent miscarriage that is corroborated by data reported in the literature. Abnormal venous hemodynamic function can be improved before conception, and this opens a new and currently unexplored pathway in the management of recurrent miscarriage.

## 1. Introduction

Recurrent miscarriage, defined as the loss of two or more consecutive pregnancies [1], affects about 2% of the population but it still remains a significant medical and emotional burden, often leaving both patients and clinicians searching for answers. While common causes such as genetic, immunological, and structural uterine abnormalities and endometrial dysfunction are well documented, an emerging area of interest is the role of maternal hemodynamics [2], particularly in regard to the function of the venous system [3].

Recent studies suggest that alterations in uterine circulation may interfere with embryo implantation or proper pregnancy progression [4,5]. The venous system plays a pivotal role in ensuring an optimal environment for embryo implantation and fetal development. Proper venous return from the pelvic region is critical for maintaining adequate blood flow to the uterus and placenta, facilitating the oxygen and nutrient exchange necessary for a healthy pregnancy [6]. However, when this balance is disrupted, either through mechanical obstruction, venous hypertension, or hypercoagulability, the resulting hemodynamic changes can lead to hypoxia, inflammation, and endothelial dysfunction—conditions known to compromise placental development. Venous dysfunction has manifested in gestational complications as pree-eclampsia [7,8]; however, its role in recurrent miscarriage remains less explored.

In this article, we focus on the role of venous circulation in recurrent miscarriage, aiming to shed light on how venous hemodynamic alterations might contribute to this incompletely understood condition.

## 2. Materials and Methods

### 2.1. Study Design

We performed a retrospective search of hospital records for data on women with a history of recurrent pregnancy loss (RPL) who underwent diagnostic evaluation at Ziekenhuis Oost-Limburg (ZOL, Genk, Belgium) and who had also a maternal cardiovascular assessment, as part of the ongoing Hasselt University Research Project of Maternal Venous Hemodynamics (medical ethical committee reference: 06/043, 08/049, 13/090U, Z 2021055).

We included the data of patients assessed between January 2022 and December 2024. The inclusion criteria were at least two consecutive pregnancy losses before 20 weeks of gestation without an underlying diagnosis after a the histology of the products of conception the histology of the products used for conception; maternal and paternal cytogenetics; hysteroscopic evaluation of the uterine cavity; and assessment of thrombophilic, endocrine, infectious, and immunologic dysfunctions [9]. Data on medical history, obstetric history, and lifestyle factors were collected through structured interviews and medical record reviews.

According to the previous study protocol reported elsewhere, all patients underwent sphygmomanometric blood pressure measurement [10], impedance cardiography assessment [10,11,12], and bio-impedance estimation of body water homeostasis [13] prior to venous Doppler assessment. In this paper, we focus on the venous Doppler measurements only.

### 2.2. Venous Doppler Sonography

All participants underwent a conventional ultrasound examination along with a combined electrocardiogram (ECG)-Doppler flow assessment of both the kidneys and liver, in a supine position, using a 3.5–7 MHz probe (Toshiba Aplio MX, Toshiba Medical Systems Belgium, Woluwe, Belgium).

The kidneys were scanned in the transverse plane just above the renal hilum, while the liver was assessed at its craniocaudal midportion, and color Doppler imaging was utilized to identify renal interlobar arteries and veins, as well as hepatic and portal venous structures.

Patients were asked to stop breathing movements during the assessment so as not to interfere with the waveform of venous flow.

Three consecutive measurements were obtained for each kidney and for the liver (left, middle, and right branches of the hepatic venous system).

The venous maximum velocity (MxV) and minimum velocity (MnV) were recorded, and the Venous Impedance Index (VI), an equivalent of the arterial Resistivity Index (RI), was calculated as [MxV − MnV]/MxV.

The time interval between the maternal ECG wave and the corresponding venous Doppler waveform characteristic was measured as the Venous Pulse Transit Time (VPTT). VPTT was corrected for heart rate, determined by the interval between two consecutive ECG R-waves.

The mean values of three measurements per organ were calculated and compared against reference ranges derived from longitudinal studies, as reported elsewhere [12]. VPTT values below the 25th normal percentile were considered abnormal. Similarly, VI values above the 75th normal percentile were considered abnormal.

**Figure 1:**
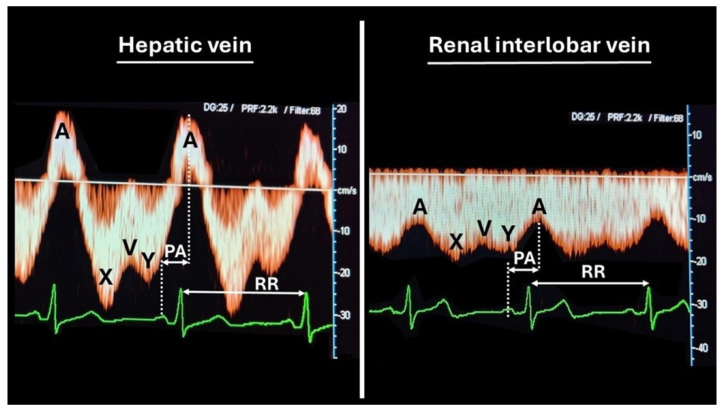
The venous Doppler sonography methodology used in this study. Venous Doppler sonography is performed on the low flow velocity spectrum with an unadjusted beam direction, using a 1.5–6 MHz convex ultrasound probe (Toshiba Aplio MX©). With the subject lying in a supine position, the liver and kidneys are scanned transversely at the midlevel plane, and three images per organ are taken as breath is held and stored. The velocities of the A, X, V, and Y deflections are measured offline. A represents the retrograde rebound of atrial contraction into the venous compartment, causing reversed flow in the liver and decelerated forward venous flow in the kidneys. X is the forward venous flow resulting from atrial diastole. V represents the decelerating forward flow during full atrial filling before the opening of the tricuspid. Y is the forward venous flow following ventricular diastole with an open tricuspid. The time interval between the ECG-P wave and Doppler A-wave is measured, as well as the duration of the cardiac cycle (RR). The venous impedance index is calculated as (X − A)/X and venous transit time as |AP|/|RR|. The venous impedance index is considered the Doppler equivalent of the atrial resistance index, rising with each increase in venous vascular tone. The venous pulse transit time shortens with an increasing venous vascular tone. The average of three values per organ is calculated and registered. A venous impedance index >75th normal percentile and a venous transit time <25th normal percentile are considered abnormal. Reference values are obtained from non-pregnant healthy young women with normal cardiovascular profiles, as previously reported [14].

Technical and methodological details are illustrated in Figure 1.

### 2.3. Literature Search

A comprehensive literature review adhering to STROBE guidelines was performed using the PubMed database. The search was performed using the advanced search mode, combining the following keywords: “Recurrent Pregnancy Loss”, “Recurrent Miscarriage”, “Vascular Function”, and “Venous Reserve”, with filters applied to titles and abstracts. No time restrictions were applied to the search. Because of a poor harvest, a second search was performed using the terms “venous capacitance”, “venous vascular function” and “low plasma volume”. The inclusion criteria consisted of original research articles published in peer-reviewed journals and written in English, focusing on the relationship between recurrent pregnancy loss and vascular or venous function. The exclusion criteria included all studies not directly addressing the topic.

## 3. Results

Nine women with a history of recurrent early pregnancy loss and a concurrent preconceptional hemodynamic evaluation were found. Among these, one woman had a history of seven miscarriages, one had a history of five, one had four, five women had three, and another one had only two early pregnancy losses. Table 1 presents the demographic characteristics of the study population.

All women were of European ethnicity, one was Mediterranean and one was East European, the others West European. The mean maternal age was higher than the 31.2 ± 4.7 years reported for Flanders’ general population in 2023, and the BMI was not different from the 2023 national mean value of 25.2 ± 5.1 [15]. All women were referred for the indication of recurrent miscarriage. Three of them did not have any other medical or obstetric antecedent. For the others, comorbidities and/or obstetric history are listed in Table 1.

Among these patients, only two had chronic conditions (hypertension and hypothyroidism). Notably, four of the nine included women (44.44%) were born with a low birth weight (<3000 g). One patient declined to complete the full investigation for recurrent miscarriage. Among the remaining eight, two were diagnosed with thrombophilia (heterozygous mutations in factor V and high factor VIII levels), while six tested negative for all assessments.

Since the association between these thrombophilia mutations and recurrent miscarriage is well established [16,17]—and given the presence of potential confounding factors in the patient who did not complete the full evaluation—we grouped only those patients whose investigation results were negative. Table 2 shows the venous hemodynamic assessment of the six cases analyzed.

Patients 1 and 6 exhibited venous circulation dysfunction both at the renal level bilaterally and in the hepatic region, as evidenced by lower VPTT values in all three locations and an elevated kidney RIVI.

Patients 3 and 5 showed less severe venous dysfunction, affecting only the right kidney.

Finally, patients 2 and 4 displayed no direct signs of venous dysfunction.

Due to the high incidence of venous dysfunction in this population, we subsequently performed a hemodynamic evaluation of the three patients without negative investigation results for recurrent miscarriage.

Table 3 presents the venous circulation assessment of these three patients.

Similarly, two out of three (66.67%) also displayed direct signs of venous dysfunction.

## 4. Discussion

The main findings of this case series are as follows: (1) abnormalities in venous circulation do not only occur during pregnancy, but can also present during the preconception phase; (2) venous dysfunction has a high prevalence in women with recurrent miscarriage, as evidenced by our evaluation of nine patients, six (66%) of whom showed such abnormalities.

To further investigate the current data on the correlation between the dysfunction of maternal venous circulation and the risk of recurrent miscarriage, and to compare the presented cases with the current knowledge, in February 2025 a literature review was performed, as previously explained. During our review we found 285 papers, but only one article specifically focused on this correlation. In this original article Donckers et al. [3] showed that women with recurrent pregnancy loss had a lower venous reserve when compared with controls, verified by a lower venous compliance in the forearm and calf and a lower plasma volume. Since there was only one article demonstrating this correlation, we found support for our observation by looking for a correlation between low plasma volume and recurrent miscarriage [2]. Indeed, it is well known that the venous system serves as a capacitance reservoir, accommodating approximately 65–70% of the total blood volume, and poor venous function is closely linked to a low plasma volume [18]. Krabbendam et al. illustrated the link between low plasma volume and poor venous reserve capacity by the induction of presyncope or higher heart rate in response to head-up tilt in women with a history of pre-eclampsia or recurrent miscarriage [19], an observation supported by a blunted autonomic nervous response after intravenous volume load in women with a low plasma volume [20]. These observations explain the earlier reported reduced capacity to increase stroke volume and venous return with prolonged moderate exercise [21]. The same research group also reported that a low plasma volume can predispose women to not only a higher recurrence of gestational hypertension disorders [22] and poor fetal growth [23], but also to a higher incidence of recurrent miscarriage [2].

**Figure 2:**
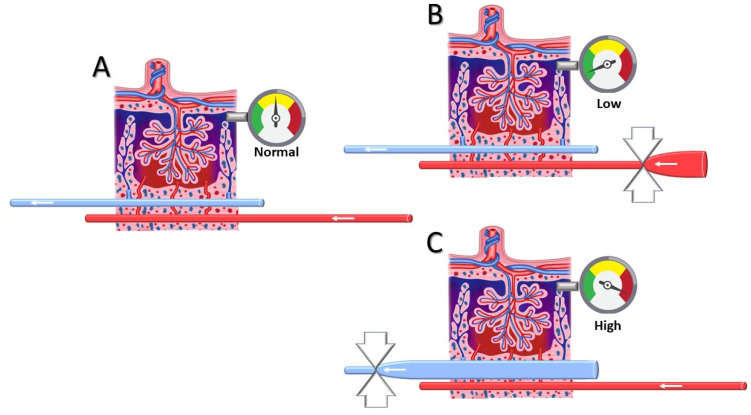
A representation of microcirculatory pressure regulation in the intervillous space. (**A**) Normal intervillous space pressure during balanced arterial supply and venous outflow. (**B**) Reduced arterial perfusion of the intervillous space from poor spiral artery dilatation is responsible for reduced pressure in the intervillous space. (**C**) Hampered venous drainage from the decidua is responsible for venous congestion with increased pressure in the intervillous space.

From a pathophysiological point of view, how can venous circulation be linked to an increased risk of miscarriage? One supportive argument is found in histology studies of the placental bed, where it is shown that between the fifth and sixth week of gestation, trophoblasts invade functional veins and lymphatic vessels at a stage where spiral artery lumina are blocked with trophoblast plugs until 10 weeks [24,25,26,27,28]. During this process, the decidual veins are permanently in direct communication with the systemic veins and, thus, are subject to abnormal venous hemodynamic function that is already present before conception [29,30]. As explained above, hampered venous drainage is responsible for increased pressure upstream at the microcirculatory level [11]. In vitro perfusion models of human placenta have shown that increasing the perfusion pressure in the intervillous space leads to morphological placental damage, vacuolization, and shedding of the syncytiotrophoblast, similar to the histologic features of pre-eclampsia [31]. As is shown in Figure 2, inadequate spiral artery adaptation with shallow dilatation is not compatible with a pressure rise downstream in the intervillous space, in contrast to the effect of congestion caused by poor venous outflow. These observations support the theory that abnormal spiral artery remodeling is a consequence, rather than a cause, of abnormal perfusion of the IVS, which may depend on pre-existing venous dysfunction [32].

The potential link between venous dysfunction and recurrent miscarriage is also supported from an indirect point of view—that is, its association with future obstetrics outcomes and late-onset cardiovascular disease. Indeed, pregnancies following an isolated miscarriage have a increased risk of complications linked to vascular abnormalities and trophoblast dysfunction [33,34], such as premature rupture of membranes, preterm delivery, and pre-eclampsia [35]. As the number of previous miscarriages increases, so does the risk of placental abruption, placenta previa, and fetal growth restriction [36]. Venous dysfunction can also contribute to the growing body of evidence linking recurrent miscarriage to an increased risk of chronic disease later in life: women who experience pre-eclampsia, early miscarriage, or recurrent miscarriage are at an increased risk of developing coronary disease in later life than women without these conditions [37,38], with a particularly higher risk in women with multiple miscarriages or stillbirths [39].

Our observation of a high rate of pre-existing maternal venous hemodynamic dysfunction in women with unexplained recurrent miscarriage opens the door to new management possibilities for women with recurrent miscarriage, aiming for the optimization of cardiovascular function before conception. Dreesen P. et al. [40] reported an improved overall cardiovascular performance after preconception physical exercise in women at risk of gestational hypertensive disorders: after an average of 29.8 weeks of exercise, participants showed a reduction in total peripheral resistances, diastolic blood pressure, and mean arterial pressure, accompanied by improvements in hepatic hemodynamics and central arterial function. Recently, the roles of some venous vasodilator drugs have also begun to garner interest, such as isosorbide dinitrate, which has been proven to enhance uterine and ovarian perfusion in animal studies [41].

One of the strengths of this study is the comprehensive follow-up of all nine patients and the rigorous and meticulous protocols of the hemodynamic assessment. However, the study is limited by the small sample size, which may restrict the generalizability of the findings. Furthermore, the etiologies underlying pregnancy loss among these patients are diverse, with some potentially interacting synergistically, while others remain partially unknown. This heterogeneity complicates the interpretation of the results and suggests the need for further research to elucidate the complex mechanisms involved.

## 5. Conclusions

This article shows that venous circulatory abnormalities have a high prevalence among women with a history of recurrent early pregnancy loss, and they can be evaluated before conception. Our observation is corroborated, both directly and indirectly, by the currently limited data reported in the literature. This supports the need for further research into venous circulation during early pregnancy, as it is a modifiable factor potentially influencing pregnancy success.

## Figures and Tables

**Table 1:** Maternal demographic and clinical data. Values are expressed as numbers (percentage) or as medians (interquartile range).

	Population (n = 9)
**Age**	35 (28.5–37.5)
<30	1 (11.1%)
30–35	4 (44.4%)
>35	4 (44.4%)
**BMI**	26.6 (21.75–28.75)
20–25	4 (44.4%)
25–30	4 (44.4%)
>30	1 (11.1%)
**Previous medical history**	
Diabetes mellitus	0 (0%)
Autoimmune hypothyroidism	1 (11.1%)
Chronic hypertension	1 (11.1%)
PID	1 (11.1%)
Maternal low birth weight	4 (44.4%)
**Previous pregnancy complications**	
Pre-eclampsia	1 (11.1%)
Fetal growth restriction	2 (22.22%)
IUFD	2 (22.22%)
**Investigation**	
Negative	6 (66.66%)
Thrombophilia	2 (22.22%)
Refused	1 (11.1%)

**Table 2:** Maternal venous hemodynamic variables compared with reference ranges derived from former studies [12,14].

	Liver		Right Kidney		Left Kidney		Result
	HVI	VPTT	RIVI	VPTT	RIVI	VPTT	
**Reference p25–p75**	1.11–1.57	0.14–0.23	0.32–0.44	0.22–0.36	0.33–0.42	0.23–0.36	
Patient 1	1.38	**0.116**	**0.499**	**0.157**	**0.457**	**0.202**	Abnormal
Patient 2	0.955	0.36	0.258	0.398	0.327	0.438	
Patient 3	1.527	0.175	**0.569**	**0.182**	0.289	0.331	Abnormal
Patient 4	0.408	0.325	0.351	0.434	0.305	0.395	
Patient 5	0.576	0.272	**0.542**	0.226	0.271	0.473	Abnormal
Patient 6	1.279	**0.088**	**0.566**	**0.076**	**0.572**	**0.126**	Abnormal

The bolds are the values that clearly show an abnormality versus the normal reference range.

**Table 3:** Maternal venous hemodynamic variables, compared with reference ranges derived from longitudinal studies [12,14], in patients with other concomitant causes of miscarriages.

	Liver		Right Kidney		Left Kidney		Result
	HVI	VPTT	RIVI	VPTT	RIVI	VPTT	
**Reference p25–p75**	1.11–1.57	0.14–0.23	0.32–0.44	0.22–0.36	0.33–0.42	0.23–0.36	
Patient 7	1.261	0.212	0.41	0.443	0.274	0.499	
Patient 8	1.487	**0.119**	**0.519**	**0.185**	**0.436**	0.285	Abnormal
Patient 9	1.461	0.151	**0.498**	**0.132**	**0.476**	0.239	Abnormal

The bolds are the values that clearly show an abnormality versus the normal reference range.

## Data Availability

The raw data supporting the conclusions of this article will be made available by the authors on request.
